# Takeout or delivery? Reoperation rates in lower extremity long bone osteomyelitis treated with debridement and local antibiotic delivery systems

**DOI:** 10.1016/j.jcot.2024.102839

**Published:** 2024-11-22

**Authors:** Nolan M. Reinhart, Jackson P. Tate, Jacob S. Budin, Julianna E. Winter, Olivia C. Lee, William F. Sherman

**Affiliations:** a Department of Orthopaedic Surgery, Tulane University School of Medicine, 1415 Tulane Ave, New Orleans, LA, 70112, USA; b Department of Orthopaedic Surgery, Louisiana State University, 2000 Canal St, New Orleans, LA, 70112, USA

**Keywords:** Osteomyelitis, Debridement, Local antibiotic delivery

## Abstract

**Background:**

Early diagnosis and treatment of osteomyelitis is essential to prevent potential complications including sepsis, extensive bone resection, amputation, and death. Despite current treatment strategies for management of osteomyelitis, recurrence rates reported in the literature are upwards of 25 %. Current evidence comparing the efficacy of differing surgical treatments of osteomyelitis is inconclusive. The purpose of this study is to compare rates of re-debridement and amputation in patients who receive either debridement alone or debridement with placement of local antibiotic delivery systems as initial treatment for lower extremity long bone osteomyelitis.

**Methods:**

A retrospective cohort study was performed to investigate complication rates after surgical treatment methods for osteomyelitis of the femur and tibia. The rates of re-debridement and amputation were compared in patients who received either debridement alone or debridement with placement of local antibiotic delivery systems.

**Results:**

This study reports 73 % lower rates of re-debridement after debridement and local antibiotic delivery in tibial osteomyelitis, and 83 % lower rates of re-debridement after debridement and placement of local antibiotic delivery systems in femoral osteomyelitis compared to debridement alone. There was no significant difference in amputation rates between treatment groups for either tibial (7.4 vs 5.7 %; OR: 1.31; 95 % CI, 0.92–1.87) or femoral osteomyelitis (2.4 vs 1.4 %; OR: 1.65; 95 % CI, 0.71–4.01).

**Conclusion:**

There was a significantly decreased likelihood of re-debridement for patients who underwent initial treatment with combined debridement and placement of antibiotic delivery systems compared to debridement alone. Providers may consider this when comparing treatment options for their patients with lower extremity osteomyelitis.

## 1 Introduction

Osteomyelitis is inflammation of bone, usually caused by a microorganism which leads to destruction and necrosis. Chronic osteomyelitis develops after weeks or months and can last years with persistent infection. With this continuous inflammation comes the risk of necrosis and destruction of the bone and surrounding tissue.^1^, ^2^, ^3^ The overall incidence of osteomyelitis is heavily dependent on risk factors such as diabetes mellitus, renal disease, peripheral vascular disease, peripheral neuropathy; however, it is estimated that the current incidence is approximately 21.8 cases per 100,000 person-years.^4^ The incidence of osteomyelitis has been on the rise in recent years due to increasing risk factors in the general population.^2^

Early diagnosis and treatment of osteomyelitis is essential to prevent potential complications including sepsis, extensive bone resection, amputation, and death. Current treatment standards for chronic osteomyelitis include a multimodal approach aimed at eradicating diseased tissue via thorough debridement and appropriate antibiotic coverage.^3^^,^^5^, ^6^, ^7^ Depending on the severity of disease and the overall health of the patient, treatment may be directed towards non-operative management with appropriate systemic antibiotics, surgical debridement of necrotic tissue, placement of local antibiotic delivery systems such as beads, cement, antibiotic powder, intramedullary nails, or any combination of these as deemed appropriate to eradicate the source of infection and prevent further loss of bone.^3^^,^^8^ Despite current treatment strategies, recurrence rates reported in the literature are upwards of 25 %.^9^

There is a paucity of data available on the outcomes of different treatment modalities for osteomyelitis in the adult population.^10^^,^^11^ The present study aims to compare the rates of complications between patients who receive surgical debridement alone or combined debridement and placement of local antibiotic delivery systems for treatment of lower extremity long bone osteomyelitis. It is hypothesized that patients who undergo surgical debridement in combination with placement of local antibiotic delivery systems will have the lower rates of subsequent debridement and amputation compared to debridement alone.

## 2 Methods

### 2.1 Data source and study design

Patient records were queried from the PearlDiver Mariner database (PearlDiver Inc., Colorado Springs, CO), a commercially available administrative claims database which contains deidentified patient data from the inpatient and outpatient settings. The database contains the medical records of patients across the U.S. from 2010 through Q3 of 2022 which are collected by an independent data aggregator. This study utilized the “M165Ortho” data set within PearlDiver, which contains 165 million patients. All health insurance payors are represented, including commercial, private, and government plans. Researchers extract data using Current Procedural Terminology (CPT) and International Classification of Diseases, Ninth and Tenth revision (ICD-9/ICD-10) codes. Institutional review board exemption was granted as provided data were de-identified and compliant with the Health Insurance Portability and Accountability Act. No outside funding was received for this study.

A retrospective cohort study was performed to investigate patient outcomes after various treatment methods for osteomyelitis of the tibia and femur. Patients who had tibial or femoral osteomyelitis were identified within the database. These patients were then filtered by initial treatment method into either debridement alone or debridement and placement of antibiotic delivery systems such as antibiotic impregnated beads, cement, powder, and antibiotic coated intramedullary nails. The rates of complications such as re-debridement and amputation were compared in these patient cohorts. Osteomyelitis of the tibia and femur were defined with ICD-10 diagnosis codes. Debridement, placement of local antibiotic delivery systems, and amputation were defined with CPT codes. Patients aged 18 to 100 were included. Patients were filtered to exclude prior diagnosis of sepsis, prior debridement, placement of local antibiotic delivery system, and amputation. All codes used to define osteomyelitis, debridement, placement of antibiotic delivery systems, amputation are provided in Appendix A.

### 2.2 Demographic data and clinical characteristics

Baseline demographic data were obtained for all patient cohorts, including age and sex. Clinical characteristics obtained included the prevalence of diabetes mellitus, obesity, tobacco use, hypertension, chronic obstructive pulmonary disease, chronic kidney disease, congestive heart failure, coronary artery disease, peripheral vascular disease, rheumatoid arthritis, and Elixhauser Comorbidity Index (ECI) (Table 1). Codes used to define clinical characteristics are provided in Appendix B.

### 2.3 Outcomes

The outcomes investigated in this study include the frequency with which patients underwent re-debridement or amputation after initial treatment for tibial or femoral osteomyelitis.

### 2.4 Statistical analysis

Statistical analyses were performed using the R statistical software (Version 4.1.0; R Project for Statistical Computing, Vienna, Austria) integrated within the PearlDiver software with an α of set to 0.05. To reduce confounding bias, propensity score matching was utilized with a caliper of 0.20 to create comparable patient cohorts. Within each group—femoral osteomyelitis, tibial osteomyelitis, and combined femoral and tibial osteomyelitis—patients treated with local antibiotic delivery plus debridement were matched in a 1:1 ratio with control patients treated with debridement alone. The matching criteria included age, sex, ECI, obesity, diabetes mellitus, tobacco use, renal failure, severe liver disease, HIV, and peripheral vascular disease.

Categorical variables were compared with a chi-square test, and continuous variables were compared with Welch's *t*-test or the Mann-Whitney *U* test. Rates of debridement and amputation after debridement or debridement and placement of local antibiotic delivery systems for treatment of osteomyelitis were compared using multivariable logistic regression adjusting for age, sex, diabetes, obesity, hypertension, COPD, chronic kidney disease, chronic heart failure, coronary artery disease, rheumatoid arthritis, and ECI. Odds ratios (ORs) with corresponding 95 % confidence intervals (CIs) were calculated for each comparison.

## 3 Results

Within 2 years after treatment of tibial osteomyelitis, compared to controls, debridement with antibiotic delivery systems demonstrated significantly lower rates of re-debridement (7.4 vs 22.2 %; odds ratio (OR): 0.27; 95 % confidence interval (CI): 0.20–0.35). Table 2. Within 2 years following treatment of femoral osteomyelitis, debridement with antibiotic delivery systems demonstrated significantly lower rates of re-debridement compared to debridement alone (5.6 vs 24.4 %; OR: 0.17; 95 % CI: 0.11–0.25).

**Table 1:** Demographics for patients with lower extremity long bone osteomyelitis.

	Tibia					Femur					Combined				
	Debridement (n = 1073)		Debridement and Antibiotic Delivery (n = 1073)			Debridement (n = 627)		Debridement and Antibiotic Delivery (n = 627)			Debridement (n = 1659)		Debridement and Antibiotic Delivery (n = 1659)		
*Complication*	*count*	*%*	*count*	*%*	*p-value*	*count*	*%*	*count*	*%*	*p-value*	*count*	*%*	*count*	*%*	*p-value*
Mean age in years (range)	56 (18–82)	–	57 (18–82)	–	0.856	55 (18–82)	–	56 (18–82)	–	0.485	56 (18–82)	–	56 (18–82)	–	0.581
Women	427	39.8	447	41.7	0.404	263	41.9	267	42.6	0.864	693	41.8	705	42.5	0.699
ECI, mean ± SD	6.3 ± 4.0	–	6.4 ± 4.0	–	0.376	6.59 ± 3.8	–	6.66 ± 4.2	–	0.736	6.46 ± 4.0	–	6.51 ± 4.1	–	0.712
Obesity	561	52.3	575	53.6	0.574	290	46.3	288	45.9	0.955	843	50.8	865	52.1	0.466
Diabetes	666	62.1	644	60.0	0.353	347	55.3	354	56.5	0.733	982	59.2	969	58.4	0.672
Tobacco Use	592	55.2	592	55.2	1.000	340	54.2	335	53.4	0.821	912	55.0	919	55.4	0.834
Hypertension	904	84.2	915	85.3	0.548	495	78.9	512	81.7	0.256	1362	82.1	1385	83.5	0.312
COPD	480	44.7	485	45.2	0.862	268	42.7	309	49.3	0.023	735	44.3	781	47.1	0.117
Chronic Kidney Disease	373	34.8	375	34.9	0.964	202	32.2	221	35.2	0.282	572	34.5	577	34.8	0.884
Congestive Heart Failure	205	19.1	204	19.0	1.000	118	18.8	120	19.1	0.943	350	21.1	312	18.8	0.108
Coronary Artery Disease	435	40.5	421	39.2	0.567	207	33.0	233	37.2	0.139	622	37.5	634	38.2	0.694
Peripheral Vascular Disease	467	43.5	479	44.6	0.633	253	40.4	254	40.5	1	712	42.9	751	45.3	0.848
Rheumatoid Arthritis	52	4.8	101	9.4	<0.0001	33	5.3	61	9.7	0.004	90	5.4	159	9.6	<0.001

**Table 2:** Reoperation rates after initial surgical management of lower extremity long bone osteomyelitis.

	Tibia					Femur					Combined				
	Debridement (n = 1073)		Debridement and Antibiotic Delivery (n = 1073)		Statistical Analysis (Ref Group, Debridement Cohort	Debridement (n = 627)		Debridement and Antibiotic Delivery (n = 627)		Statistical Analysis (Ref Group, Debridement Cohort	Debridement (n = 1659)		Debridement and Antibiotic Delivery (n = 1659)		Statistical Analysis (Ref Group, Debridement Cohort
Complication	n	%	n	%	OR (95 % CI)	n	%	n	%	OR (95 % CI)	n	%	n	%	OR (95 % CI)
Re-debridement	238	22.2	79	7.4	0.27 (0.20–0.35)	153	24.4	35	5.6	0.17 (0.11–0.25)	390	23.5	124	7.5	0.26 (0.21–0.32)
Amputation	61	5.7	79	7.4	1.31 (0.92–1.87)	9	1.4	15	2.4	1.65 (0.71–4.01)	80	4.8	99	6.0	1.28 (0.94–1.75)
All Reoperations	282	26.3	147	13.7	0.43 (0.34–0.53)	158	25.2	47	7.5	0.23 (0.16–0.32)	448	27.0	206	12.4	0.37 (0.31–0.45)

There were no significant differences in rates of amputation between the debridement cohort and the antibiotic delivery cohort within 2 years after initial treatment of tibial osteomyelitis (7.4 vs 5.7 %; OR: 1.31; 95 % CI: 0.92–1.87) or femoral osteomyelitis (2.4 vs 1.4 %; OR: 1.65; 95 % CI: 0.71–4.01).

When examining all patients with lower extremity long bone osteomyelitis, those who underwent debridement with antibiotic delivery systems demonstrated significantly lower rates of all reoperations (12.4 vs 27.0 %; OR: 0.37; 95 % CI: 0.31–0.45), including re-debridement (7.5 vs 23.5 %; OR: 0.26; 95 % CI: 0.21–0.32), within 2 years of initial surgical treatment. There was no significant difference in amputations between treatment groups (6.0 vs 4.8 %; OR: 1.28; 95 % CI: 0.94–1.75).

## 4 Discussion

Reoperations for recurrence and persistence of infection in the setting of osteomyelitis can lead to significant morbidity and mortality.^2^ Lang et al. found that up to 26 % of patients treated with debridement alone for chronic osteomyelitis required additional operations to eradicate infection, although this heavily depends on other risk factors such as age and smoking status.^12^ The addition of local antibiotic delivery systems such as antibiotic impregnated beads, cement, powder, and intramedullary nails have become commonplace in the treatment of more severe osteomyelitis, as it has been shown to lead to early suppression of infection, greater than two-fold reduction in recurrence of osteomyelitis and minimization of pathologic fracture risk due to internal reinforcement with use of antibiotic coated IM nails.^13^, ^14^, ^15^, ^16^ The results presented here further highlight the efficacy of debridement augmentation with local antibiotic delivery system placement for the treatment of lower extremity long bone osteomyelitis to minimize adverse outcomes such as re-debridement and amputation.

Local antibiotic placement has been shown to bolster the efficacy of debridement in patients with osteomyelitis. McNally et al. report a 6 % infection recurrence rate at a mean follow-up of 6 years in patients with Cierny-Mader Types III and IV chronic osteomyelitis treated with a single stage debridement and placement of local antibiotics.^17^ Similarly, this study demonstrated a significantly lower rate of re-debridement in patients who underwent initial debridement and insertion of local antibiotic delivery systems compared to those who received debridement alone. Prior studies also found similar results, reporting as low as a 0 % reoperation rate for combined debridement and local antibiotic delivery systems.^10^^,^^12^^,^^18^^,^^19^ Increased concentrations of antibiotics at the site of infection may influence these results, as local antibiotics avoid the poor bioavailability in bone and diseased tissue that limits the efficacy of systemic antibiotics in these patients.^14^^,^^20^

Despite lower rates of re-debridement following local antibiotic delivery, there was no difference in amputation rates between the debridement only and local antibiotic delivery cohorts. Similarly, a retrospective cohort study performed by Qin et al., in 2019 reported that use of antibiotic impregnated calcium sulfate beads in the treatment of diabetic foot osteomyelitis did not reduce post-operative amputation rates compared to debridement alone, and they concluded that this is possibly a result of a short study period.^21^ Likewise, the results seen here may be due to a relatively short follow-up of 2 years. Procedures such as amputation are considered last resort options for limb salvage after devastating injuries or disease and are associated with significant and permanent disability. It is possible that many patients and surgeons pursue limb sparing options for longer than 2 years from their initial treatment until amputation becomes absolutely necessary.^22^^,^^23^

These findings should be interpreted in the context of this study's limitations. By only evaluating complications within 2 years, this analysis is limited to short term outcomes. Furthermore, because continuous database enrollment for 2 years after initial treatment for osteomyelitis was required for inclusion, patients who died within 2 years after initial surgery were excluded. Therefore, these results may not be applicable to patients who have high perioperative mortality risk. The potential of coding errors is inherent with any analysis of administrative claims data. However, such instances are infrequent and made up only 0.9 % of Medicare and Medicaid payments in 2023.^24^ Given the retrospective nature of this study, it is hard to establish a causal relationship between complication rates and treatment modality. Severity of osteomyelitis could not be determined, as diagnosis codes do not have linked Cierny-Mader classifications. Due to poor specificity of some local drug delivery codes, it is possible that the local antibiotic delivery cohort may include patients who received local placement of drugs other than antibiotics. Treatment decisions are intimately tied to severity of disease, so the results may misrepresent the severity of disease in some cases by using subsequent treatments as a surrogate for outcomes. Additionally, osteomyelitis recurrence could not be determined, as diagnosis codes do not indicate resolution or recurrence of disease, only that a disease has been present or not. This study spans 2010 to 2022, in that time many advancements have been made in local antibiotic delivery systems, therefore the true impact of these treatment modalities may not have been fully captured in this study.^25^, ^26^, ^27^

In this retrospective cohort analysis, patients who underwent combined debridement with local antibiotic delivery demonstrated an 83 % reduction in likelihood of re-debridement compared to controls. Providers may consider this when comparing surgical treatment options for their patients with lower extremity osteomyelitis.

## Declaration of competing interest

The authors declare that they have no known competing financial interests or personal relationships that could have appeared to influence the work reported in this paper.

## Figures and Tables

**Table:** 

Diagnosis/Procedure	ICD 10 and CPT Codes
Tibial Osteomyelitis	ICD-10-D-M86061, ICD-10-D-M86062, ICD-10-D-M86069, ICD-10-D-M86161, ICD-10-D-M86162, ICD-10-D-M86169, ICD-10-D-M86261, ICD-10-D-M86262, ICD-10-D-M86269, ICD-10-D-M86361, ICD-10-D-M86362, ICD-10-D-M86369, ICD-10-D-M86461, ICD-10-D-M86462, ICD-10-D-M86469, ICD-10-D-M86561, ICD-10-D-M86562, ICD-10-D-M86569, ICD-10-D-M86661, ICD-10-D-M86662, ICD-10-D-M86669, ICD-10-D-M868X6
Femoral Osteomyelitis	ICD-10-D-M86051, ICD-10-D-M86052, ICD-10-D-M86059, ICD-10-D-M86151, ICD-10-D-M86152, ICD-10-D-M86159, ICD-10-D-M86251, ICD-10-D-M86252, ICD-10-D-M86259, ICD-10-D-M86351, ICD-10-D-M86352, ICD-10-D-M86359, ICD-10-D-M86451, ICD-10-D-M86452, ICD-10-D-M86459, ICD-10-D-M86551, ICD-10-D-M86552, ICD-10-D-M86559, ICD-10-D-M86651, ICD-10-D-M86652, ICD-10-D-M86659, ICD-10-D-M868X5
Debridement	CPT-11012, CPT-11044, CPT-27360, CPT-27640
Local Antibiotic Delivery	CPT-11981, CPT-11982, CPT-11983, CPT-G0516, CPT-20700, CPT-20701, CPT-20702, CPT-20703, CPT-20704, CPT-20705
Amputation	CPT-27882, CPT-27596, CPT-27881, CPT-27880, CPT- 27594, CPT-27591, CPT-27592, CPT-27590, CPT-27888, CPT-27886, CPT-27884
Sepsis	ICD-9-D-99591, ICD-9-D-99592, ICD-9-D-78552, ICD-10-D-R6520, ICD-10-D-R6521, ICD-10-D-T8144XA, ICD-10-D-T8144XD, ICD-10-D-T8144XS, ICD-10-D-A419, ICD-10-D-A4189, ICD-10-D-A412, ICD-10-D-A411, ICD-10-D-A4102, ICD-10-D-A4101, ICD-10-D-A409, ICD-10-D-A408, ICD-10-D-A403, ICD-10-D-A400

**Table:** 

Demographic	ICD 9/10 Codes
Obesity	ICD-9-D-2780, ICD-9-D-27800, ICD-9-D-27801, ICD-9-D-27802, ICD-9-D-27803, ICD-10-D-E660:ICD-10-D-E669
Diabetes	ICD-9-D-24900:ICD-9-D-25099, ICD-9-D-7902, ICD-9-D-79021, ICD-9-D-79022, ICD9-D-79029, ICD-9-D-7915, ICD-9-D-7916, ICD-10-D-E090:ICD-10-D-E139
Tobacco Use	ICD-9-D-3051, ICD-9-D-V1582, ICD-10-D-F17220, ICD-10-D-F17221, ICD-10-D-F17223, ICD-10-D-F17228, ICD-10-D-F17229, ICD-10-D-F17290, ICD-10-D-F17291, ICD-10-D-F17293, ICD-10-D-F17298, ICD-10-D-F17299, ICD-10-D-Z720
Hypertension	ICD-9-D-4010:ICD-9-D-4059, ICD-10-D-I10:ICD-10-D-I159
COPD	ICD-9-D-490:ICD-9-D-49699, ICD-10-D-J44:ICD-10-DJ449
Chronic Kidney Disease	ICD-9-D-585, ICD-9-D-5851, ICD-9-D-5852, ICD-9-D-5853, ICD-9-D5854, ICD-9-D-5855, ICD-9-D-5856, ICD-9-D-5859, ICD-9-D-7925, ICD-10-D-N18:ICD-10-D-N189
Congestive Heart Failure	ICD-9-D-39891, ICD-9-D-4280, ICD-9-D-4281, ICD-9-D-42820, ICD-9-D-42821, ICD-9-D-42822, ICD-9-D-42823, ICD-9-D-42830, ICD-9-D-42831, ICD-9-D-42832, ICD-9-D-42833, ICD9-D-42840, ICD-9-D-42841, ICD-9-D-42842, ICD-9-D-42843, ICD-9-D-4289, ICD-10-D-I150:ICD-10-D-I159
Coronary Artery Disease	ICD-9-D-4110:ICD-9-D-4149, ICD-10-D-I25:ICD-10-D-I259
Peripheral Vascular Disease	ICD-9-D-093, ICD-9-D-4373, ICD-9-D-440:ICD-9-D-44099, ICD-9-D-441:ICD-9-D-44199, ICD-9-D-4431:ICD-9-D-4439, ICD-9-D-4471, ICD-9-D-5571, ICD-9-D-5579, ICD-9-D-V434, ICD-10-DI70:ICD-10-D-I7099, ICD-10-D-I71:ICD-10-D-I7199, ICD-10-D-I731, ICD-10-D-I738, ICD-10-D-I739, ICD-10-D-I771, ICD-10-D-I790, ICD-10-D-I792, ICD-10-D-K551, ICD-10-D-K558, ICD-10-D-K559, ICD-10-Z958, ICD10-D-Z959
Rheumatoid Arthritis	ICD-9-D-7140, ICD-9-D-7142, ICD-10-M0520:ICD-10-D-M061
